# Maternal exposure to ambient air pollution and fetal growth in North-East Scotland: A population-based study using routine ultrasound scans

**DOI:** 10.1016/j.envint.2017.07.018

**Published:** 2017-10

**Authors:** Tom Clemens, Steve Turner, Chris Dibben

**Affiliations:** a School of Geosciences, University of Edinburgh, Edinburgh, Scotland, UK; b Child Health, University of Aberdeen, Aberdeen, Scotland, UK

**Keywords:** Fetal growth, Ambient air pollution, Maternal health, In utero, Scotland

## Abstract

**Background:**

Maternal ambient air pollution exposure is associated with reduced birthweight. Few studies have examined the effect on growth in utero and none have examined the effect of exposure to particulates less than 2.5 µm (PM_2.5_) and possible effect modification by smoking status.

**Objectives:**

Examine the effect of maternal exposure to ambient concentrations of PM_10_, PM_2.5_ and nitrogen dioxide (NO_2_) for in utero fetal growth, size at birth and effect modification by smoking status.

**Methods:**

Administratively acquired second and third trimester fetal measurements (bi-parietal diameter, femur length and abdominal circumference), birth outcomes (weight, crown heel length and occipito-frontal circumference) and maternal details were obtained from routine fetal ultrasound scans and maternity records (period 1994–2009). These were modelled against residential annual pollution concentrations (calendar year mean) adjusting for covariates and stratifying by smoking status.

**Results:**

In the whole sample (*n* = 13,775 pregnancies), exposure to PM_10_, PM_2.5_ and NO_2_ was associated with reductions in measurements at birth and biparietal diameter from late second trimester onwards. Among mothers who did not smoke at all during pregnancy (*n* = 11,075), associations between biparietal diameter and pollution exposure remained significant but were insignificant among those who did smoke (*n* = 2700). Femur length and abdominal circumference were not significantly associated with pollution exposure.

**Conclusions:**

Fetal growth is strongly associated with particulates exposure from later in second trimester onwards but the effect appears to be subsumed by smoking. Typical ambient exposures in this study were relatively low compared to other studies and given these results, it may be necessary to consider reducing recommended “safe” ambient air exposures.

## Highlights

• We examined the effect of maternal pollution exposure for fetal growth and size.
• Exposure to particulates and NO_2_ strongly associated with reductions in head growth and size.
• Effects were strongest for non-smokers.
• Pollution effects were observed despite a relatively low exposure environment.

## 1 Introduction

Air pollution is a major component of the total global burden of disease (Lim et al., 2012) associated with, for example, approximately 40,000 deaths per year and associated annual healthcare costs of around £20 billion in the UK (Royal College of Physicians, 2016). The harmful effects of ambient air pollution are apparent at birth with associations between pollution exposures, particularly fine particulate matter with a diameter less than ten microns (PM_10_) and nitrogen oxides, and adverse neonatal outcomes such as reduced birthweight, prematurity and birth head circumference (Dibben and Clemens, 2015, Hjortebjerg et al., 2016, Malley et al., 2017, Pedersen et al., 2013, Rich et al., 2015, Stieb et al., 2016). Some studies have used ultrasound scans to examine when in utero exposure to air pollution may be linked to growth restriction (Aguilera et al., 2010, Carvalho et al., 2016, Hansen et al., 2008, Iñiguez et al., 2012, Malmqvist et al., 2017, Ritz et al., 2014, Slama et al., 2009, van den Hooven et al., 2012). Collectively these studies show some evidence of an association between increased maternal exposures and reduced fetal head size however there are limitations with some of these studies and also variations in methodology that may be important. Some of the studies are based on fairly small sample sizes and there are potential differences in how pollution exposure is assigned. One study used, for example, a nearest static monitor approach (Hansen et al., 2008), which may introduce a bias to the null hypothesis (Butland et al., 2013), and others use modelled concentrations from land use regression techniques(Aguilera et al., 2010, Ritz et al., 2014, van den Hooven et al., 2012). The main determinant of poor quality ambient air is combustion of fossil fuels where PM_10_, PM_2.5_ (particulates with a diameter < 2.5 μm) and nitrogen dioxide (NO_2_) arise. Importantly, of those previous studies, that have looked specifically at in utero fetal growth, only three have examined PM_10_ and NO_2_ exposure (Aguilera et al., 2010, Ritz et al., 2014, van den Hooven et al., 2012) and none have examined PM_2.5_ which may be an important determinant of birth outcomes (Sun et al., 2016).

In this paper we extend existing research by examining the effect of PM_2.5_ as well as PM_10_ and NO_2_ on in utero fetal growth in the relatively low pollution environment of North-East Scotland. Using a whole population cohort of pregnancies and associated routine ultrasound sonography information from North-East Scotland, we aim to test the hypothesis that maternal ambient outdoor air exposures to increased PM_2.5_, PM_10_ and NO_2_ concentrations are associated with reduced fetal size and growth. This large cohort also allows us to examine models stratified by smoking status to determine possible differences in the effect of pollutant exposure among smokers and non-smokers.

## 2 Methods

### 2.1 Study population

We use maternal and fetal data from the Aberdeen Maternity and Neonatal Databank (AMND) which has archived routinely acquired data from clinical activity at Aberdeen Maternity Hospital (AMH) since 1950 (Ayorinde et al., 2016). This hospital is the delivery unit for approximately 80% of the population of North East Scotland and 95% of deliveries in Aberdeen City (Thompson et al., 2010). Ultrasound scan assessments started at AMH in the mid-1980s but only scans occurring in the period for which pollution data were available were used for the present analysis (2002 − 2011). Because only 1.5% of the population of North East Scotland is minority ethnic (as recorded at the 2011 census) we did not consider ethnicity in the study. Furthermore, only singleton births were considered.

### 2.2 Exposure assessment

To estimate outdoor air pollution exposure, we use modelled concentration estimates that are based on calendar year emissions totals and so represent annual average concentrations. These data are available UK wide at a spatial resolution of 1 km × 1 km from 2002 onwards for PM_2.5_, PM_10_ and NO_2_ and are supplied by the United Kingdom Department for the Environment, Food and Rural Affairs (DEFRA) (Brookes et al., 2011, NETCEN, 2005). The concentration estimates are generated from a Pollution Climate Mapping (PCM) approach which takes local and distant point and area sources and sums the annual concentration values. For NO_2,_ the National Atmospheric Emissions Inventory (*NAE*I) is used to determine concentration values for point sources. Distant sources are modelled with a combination of dispersion models and rural background static monitors. Area concentration sources are modelled with dispersion kernels and *NAE*I data (Brookes et al., 2011). Because the composition of PM pollution is more heterogeneous, different approaches are used details of which can be found in Brookes et al. (2011, pp. 17–18).

Full address location was unavailable in the dataset and so we allocate exposure values to each mother on the basis of the centroid of the postcode of residence, recorded at the time of delivery. Postcodes vary in size from single apartment blocks in urban centres (< 100 m^2^) to much larger areas in rural locations (up to around 200 km^2^ in the very sparsely populated upland areas of Western North East Scotland). However, pollution levels in rural scotland are low and spatially homogenous and therefore larger postcode areas further from urban centres are unlikely to introduce significant exposure misclassification. We use the population weighted postcode centroid coordinates and a geographical information system to determine an exposure estimate from the gridded concentration data based on the grid cell within which the centroid point is located. To adjust for unmeasured annual variation in the modelled concentrations, we include a dummy term for year of birth in the models. We calculated frequency quartiles of each pollutant and used these as well as continuous measures as exposure variables.

### 2.3 Birth outcomes, fetal ultrasound measurements and growth curves

First trimester scans (i.e. ≤ 13 weeks gestation) are typically made at 10–12 weeks gestation to determine the gestational age of the pregnancy. Second trimester scans (i.e. 13– < 28 weeks) take place close to 20 weeks gestation in order to screen for fetal anomalies. Third trimester scans (i.e. ≥ 28 weeks gestation) are conducted for obstetric indications such as breech presentation or in uterine growth retardation. Some pregnancies were scanned multiple times in each trimester. At the first trimester, crown rump length (CRL) is recorded and during second and third trimester scans abdominal circumference (AC), femur length (FL) and bi-parietal diameter (BPD) are recorded. All measurements are recorded in mm. Gestational age is estimated from maternal last menstrual period (LMP) unless there is a discrepancy of > 2 weeks between gestation from LMP and first trimester scan, in which case the latter is used as the reference. ATL (Ultramark 4A) or Toshiba (SSA-240A or SSA-340A) ultrasound scanners are used to determine fetal measurements and are calibrated in accordance with manufacturer's recommendations. The inter-observer coefficient of variation for CRL measurements is < 10% (De Biasio et al., 2002). We also examined the following outcomes measured at birth; crown heel length (CHL), occipitofrontal circumference (OFC) and birthweight.

To address the complexity of non-linear fetal growth, we estimated growth trajectories for each of the fetal characteristics by determining the best fitting second degree transformation functions of gestational age from a set of fractional polynomial powers (Royston et al., 1999). Fractional polynomials are useful for modelling non-linear growth processes because they reduce the risks of overfitting associated with non-parametric local smoothing techniques. They are more flexible than standard parametric polynomial quadratic or cubic spline approaches which are less adept at fitting to data points at extremes of the distribution and may introduce artefactual turning points or implausible shapes in the fitted curves (Tilling et al., 2014). From these models we extracted both standardised residuals at each week of gestation (standard deviation (SD) scores) and the transformed gestational age variables.

### 2.4 Cross-sectional analysis

For the cross sectional fetal size analyses we use mixed effects regression models stratified by trimester of scan (2nd or 3rd trimester) using SD scores as the outcome. Because of the clustering in the data (multiple scans for the same pregnancy in each trimester as well as mothers with more than one pregnancy) we assisgned pregnancy and mother to different levels in the model and included random intercepts for each. We adjust these models for mothers age at delivery, parental social class, parity, sex of the baby, maternal height and weight in early pregnancy, maternal smoking and the year of scan. Models were estimated treating pollutants as both categorical and continuous. In the latter, effects associated with increases of 5 μg/m^3^ for PM_2.5_ and 10 μg/m^3^ for PM_10_ and NO_2_ were reported for comparability witrh previous studies.

Birth outcomes are modelled on their original scale using ordinary least squares linear regression models with clustered errors within mothers. We adjust these models for the best fitting fractional polynomial transformation function of gestational age at birth as well as the same covariates as the cross-sectional fetal size models.

### 2.5 Longitudinal growth models

We estimated longitudinal growth models for each of the fetal measurements on their original scales using mixed effect multilevel models with an auto-regressive residual error structure. We assigned pregnancy and mother to levels of the model and estimated random intercept terms for each level. The effect of pollutants for fetal growth was estimated by including both main effects of the transformed gestational age variables and pollutant exposure (as a continuous variable scaled as above) and their interactions. We also considered both main effects and interactions with gestational age of other covariates and selected the best fitting combination based on the Bayesian Information Criterion. For all outcomes the best fitting model consisted of main effects for all covariates as well as interactions between transformed gestational age and baby sex, maternal weight and pollutant exposure.

We present results graphically, showing for each fetal characteristic, absolute growth trajectories for those with the lowest pollution exposure compared to those exposed to the maximum exposure. 95% confidence intervals are shown to highlight growth trajectories that are statistically significantly different. We also present differences in these growth trajectories expressed as differences in SD score to allow comparison across pollutants and fetal characteristics. For those fetal characteristics that are related to pollutant exposure in the main models, we also consider their interactions with smoking. We do this both by estimating models stratified by subgroups of smoking status (smokers and non-smokers) and by estimating a single model which includes three-way interactions terms between transformed gestational age, smoking and pollution exposure.

## 3 Results

### 3.1 Sample characteristics

The study included data from 13,775 pregnancies in 12,467 mothers between 2002 and 2011, the period for which complete pollution information were available. Table 1 and Table 2 show descriptive statistics for, respectively, the covariates and outcomes used in the study. Air pollution exposure information for the samples is presented in Table 3.

**Table 1:** Descriptives for maternal characteristics and fetal measurements.

N (Pregnancies)	13,775	
Categorical variables	Number	%
Parity		
Nulliparous	6731	48.86
One previous pregnancy	4820	34.99
Two previous pregnancies	1541	11.19
3 or more previous pregnancies	683	4.96
Age		
19 and under	717	5.21
20–40	12,803	92.94
Over 40	254	1.84
Offspring sex		
Female	6706	48.68
Male	7069	51.32
Smoking		
Ex smoker, non-smoker	11,075	80.40
Smoker	2700	19.60
Social class		
Professional	2193	15.92
Managerial and technical	3883	28.19
Skilled non-manual	1932	14.03
Skilled manual	1425	10.34
Partly skilled	1345	9.76
Unskilled	322	2.34
Not available	2675	19.42
Continuous variables	Mean	
Maternal height (cm)	163.75	
Maternal weight (kg)	68.60

**Table 2:** Descriptive statistics for outcomes.

Fetal characteristics trimester 2	Mean (SD in brackets)	Mean gestational age (SD in brackets)
Bi-parietal diameter (mm) (14,172 scans)	47.6 (9.3)	19.5 (2.8)
Femur length (mm) (15,417 scans)	33.0 (7.6)	19.6 (2.7)
Abdominal circumference (mm) (12,606 scans)	165.9 (31.3)	20.5 (2.8)
Fetal characteristics trimester 3		
Bi-parietal diameter (mm) (5728 scans)	84.6 (7.0)	33.3 (3.1)
Femur length (mm) (6258 scans)	64.7 (6.5)	33.5 (3.2)
Abdominal circumference (mm) (9171 scans)	296.4(35.0)	33.4 (3.2)
Birth characteristics (pregnancies in brackets)		
Crown heel length (cm) (13,667 pregnancies)	49.5 (3.0)	39.0 (2.3)
Occipitofrontal circumference (cm) (13,714 pregnancies)	34.5 (1.9)	39.0 (2.3)
Birthweight (kg) (13,756 pregnancies)	3339.8 (630.5)	39.0 (2.3)

**Table 3:** Distribution of pollutants.

									Correlations	
	N (scans)	Min	25th ptile	Mean	SD	Median	75th ptile	Max	PM10	PM25
PM_10_	139,028	6.4	11.1	12.6	2.3	12.4	14.0	19.1	1	
PM_2.5_	71,274	3.3	6.0	7.2	1.6	7.3	8.6	10.9	0.9234	1
NO_2_	138,639	1.0	6.1	13.4	8.2	12.6	20.8	38.2	0.6846	0.6365

### 3.2 Ambient air exposure and fetal antenatal measurements

**Fig. 1:**
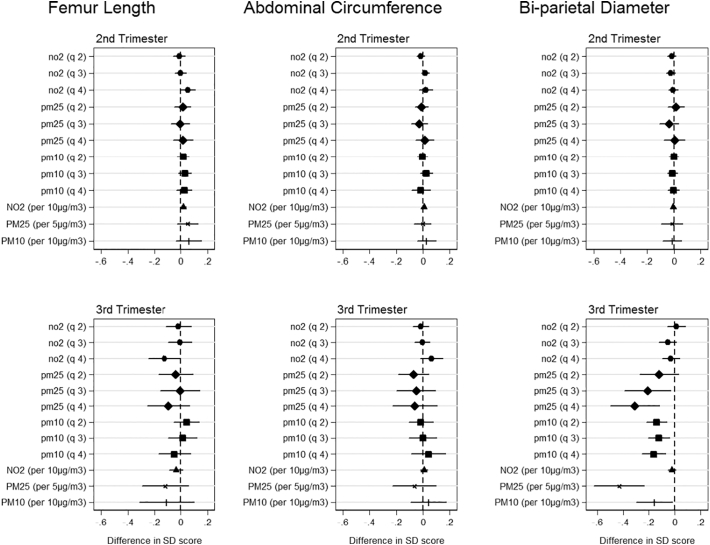
Cross-sectional trimester models of ambient air pollutant exposure and fetal size. Coefficients report change in SD score with the 2nd, 3rd and 4th pollutant quartiles relative to the 1st quartile for each fetal characteristic in 2nd and 3rd trimester. The models are adjusted for parity, age, sex, smoking, social class, maternal height and weight.

Fig. 1 shows associations between the three different pollutants (categorical (quartiles) and continuous) and fetal measurements from the trimester specific models. In the second trimester, none of the pollutants are significantly associated with a change in standard deviation score for any fetal measurements. In the third trimester, exposures are not associated with femur length and abdominal circumference. However, bi-parietal diameter in the third trimester is significantly reduced with exposure to both PM_2.5_ and PM_10_ but not NO_2_. PM_2.5_ shows an ordered gradient across the lowest to highest quartiles with quartile 4 showing reductions in biparietal diameter SD score of 0.32 (95% CI: 0.13, 0.52) compared to the lowest exposure quartile. For the coefficients for PM_10_, the highest exposure quartile also has the greatest reductions in SD score for biparietal diameter of 0.16 (95% CI: 0.06, 0.26). Continuous trends in PM_2.5_ and PM_10_ are also significantly associated with a reduction in bi-parietal diameter SD scores of − 0.43 (95% CI: − 0.63, − 0.24) and − 0.16 (95% CI: − 0.30, − 0.02) respectively. Results were consistent in sensitivity analysis when stratifying by term births and fetal sex.

### 3.3 Ambient air exposure and fetal size at birth

**Fig. 2:**
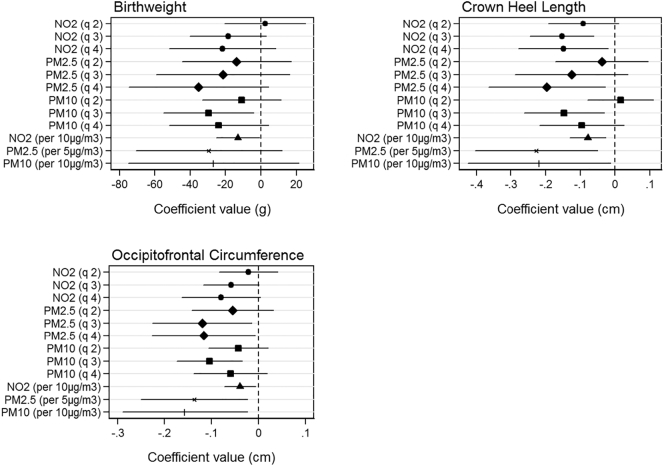
Cross-sectional models of ambient air pollutant exposure and birth size measurements. Each graph shows effect of 2nd, 3rd and 4th pollutant quartiles relative to 1st quartile. Effects are shown in grams for birthweight and centimeters for crown heel length and occipitofrontal circumference. The models are adjusted for parity, age, sex, smoking, social class, maternal height and weight.

Fig. 2 shows associations between pollutant quartiles and fetal measurements at birth. For birthweight, we find no significant relationships with exposure to PM_2.5_ or NO_2_. Quartile 3 for PM_10_ shows reduction effects on birthweight of 28.0 g (95% CI: 6.8, 49.2) compared to the lowest quartiles of exposure. For crown heel length relative to quartile 1, NO_2_ shows reductions associated with quartiles 3 and 4 of 1.4 mm (95% CI: 0.7, 2.1) and 1.5 mm (95% CI: 0.7, 2.3) respectively, PM_2.5_ for quartile 4 of 2 mm (95% CI: 0.3, 3.6) and PM_10_ for quartile 3 of 1.1 mm (95% CI: 0.2, 2.0). Continuous trends for all pollutants are significant with reductions of − 0.08 cm (95% CI: − 0.13, − 0.03), − 0.23 cm (95% CI: − 0.40, − 0.05) and − 0.22 cm (95% CI: − 0.42, − 0.01) associated with NO_2_, PM_2.5_ and PM_10_ respectively. Finally, relative to exposure quartile 1, occipitofrontal circumference shows significant reductions associated with PM_2.5_ quartiles 3 and 4 of 1.2 mm (95% CI: 0.2, 2.3) and 1.2 mm (95% CI: 0.1, 2.3) respectively and PM_10_ quartile 3 of 0.7 mm (95% CI: 0.2, 1.3). Continuous trends are also significant with reductions of − 0.04 cm (95% CI: − 0.07, − 0.01), − 0.14 cm (95% CI: − 0.25, − 0.02) and − 0.16 cm (95% CI: − 0.29, − 0.02) associated with NO_2_, PM_2.5_ and PM_10_ respectively. Results were consistent in sensitivity analysis when stratifying by term births and fetal sex.

### 3.4 Ambient air exposure and longitudinal fetal growth

**Fig. 3:**
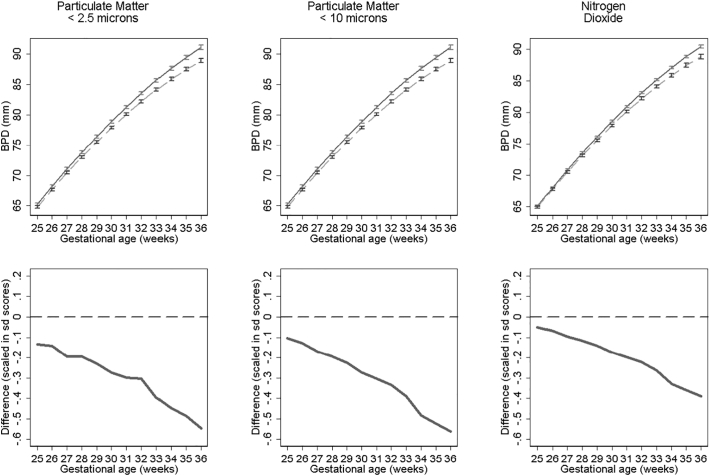
Estimated bi-parietal diameter growth trajectories and relative differences for different levels of pollutant exposure. Top panels show estimated growth trajectories for minimum exposure (solid lines) compared to maximum exposure (dashed line). Bottom panels show the difference in these trajectories expressed in units of SD scores. The models are adjusted for parity, age, sex, smoking, social class, maternal height and weight. To aid interpretation, x axis is truncated below 25 weeks.

**Fig. 4:**
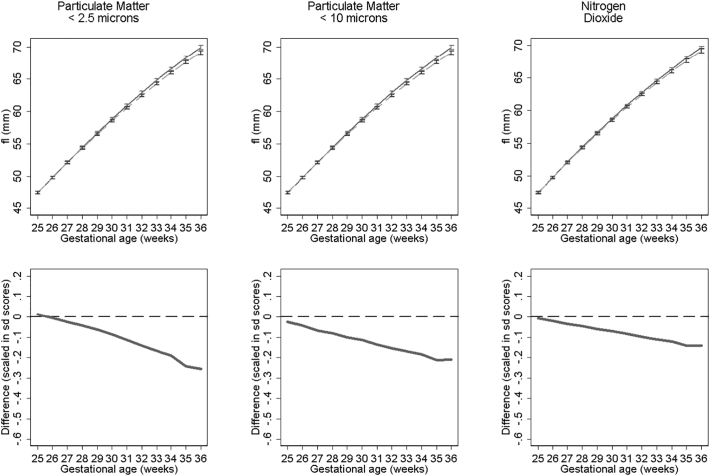
Estimated femur length growth trajectories and relative differences for different levels of pollutant exposure. Top panel shows estimated growth trajectories for minimum exposure (solid lines) compared to maximum exposure (dashed line). Bottom panel shows the difference in these trajectories expressed in units of SD scores. The models are adjusted for parity, age, sex, smoking, social class, maternal height and weight. To aid interpretation, x axis is truncated below 25 weeks.

**Fig. 5:**
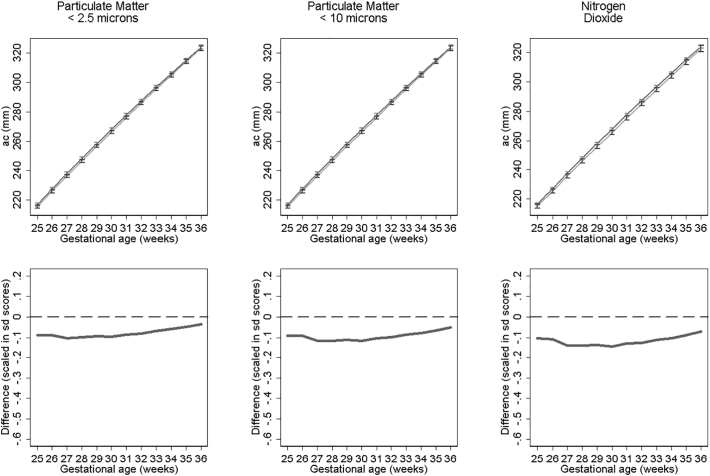
Estimated abdominal circumference growth trajectories and relative differences for different levels of pollutant exposure. Top panels show estimated growth trajectories for minimum exposure (solid lines) compared to maximum exposure (dashed line). Bottom panels show the difference in these trajectories expressed in units of SD scores. The models are adjusted for parity, age, sex, smoking, social class, maternal height and weight. To aid interpretation, x axis is truncated below 25 weeks.

Fig. 3, Fig. 4, Fig. 5 show fetal measurement growth trajectories for those exposed to the least level of pollution (solid lines) compared to those exposed to the maximum exposure (dashed lines). They also show the differences between these trajectories expressed as relative differences. For biparietal diameter (BPD) both PM_10_ and PM_2.5_ show statistically reductions from late second trimester onwards and NO_2_ from around 30 weeks onwards (Fig. 3). PM_10_ shows the biggest reductions in terms of relative differences. These reductions result in BPD's for those with the highest exposure that are 1.58 mm, 2.18 mm and 2.28 mm smaller at 36 weeks gestation than those with the lowest exposure to NO_2_, PM_2.5_ and PM_10_ respectively. Growth trajectories for femur length (Fig. 4) show small but statistically insignificant differences and abdominal circumference (Fig. 5) show no statistical significant differences between pollutant exposures. Results were consistent in sensitivity analysis when stratifying by term births and fetal sex.

### 3.5 Results stratified by smoking

**Fig. 6:**
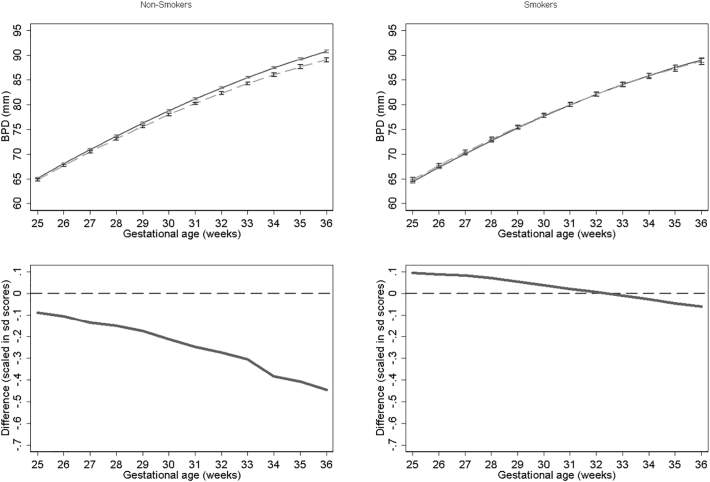
Estimated growth trajectories and relative differences for different levels of NO_2_ exposure for bi-parietal diameter stratified by maternal smoking status. Top panels show estimated growth trajectories for minimum exposure (solid lines) compared maximum exposure (dashed line). Bottom panels show the difference in these trajectories expressed in units of SD scores. The models are adjusted for parity, age, sex, smoking, social class, maternal height and weight. To aid interpretation, x axis is truncated below 25 weeks.

**Fig. 7:**
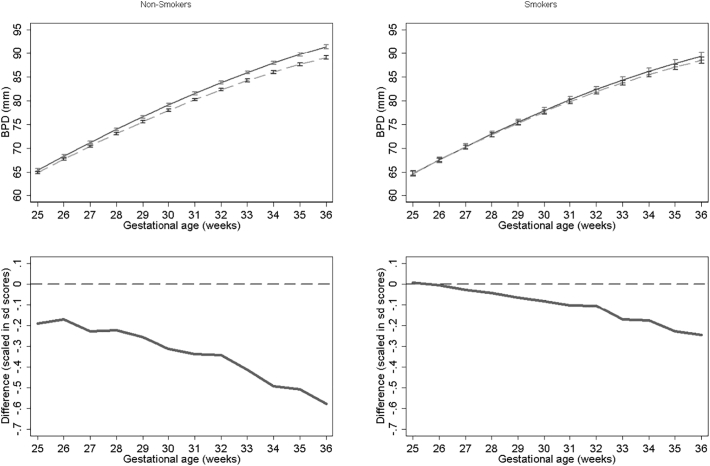
Estimated growth trajectories and relative differences for different levels of PM_2.5_ exposure for bi-parietal diameter stratified by maternal smoking status. Top panels show estimated growth trajectories for minimum exposure (solid lines) compared maximum exposure (dashed line). Bottom panels show the difference in these trajectories expressed in units of SD scores. The models are adjusted for parity, age, sex, smoking, social class, maternal height and weight. To aid interpretation, x axis is truncated below 25 weeks.

**Fig. 8:**
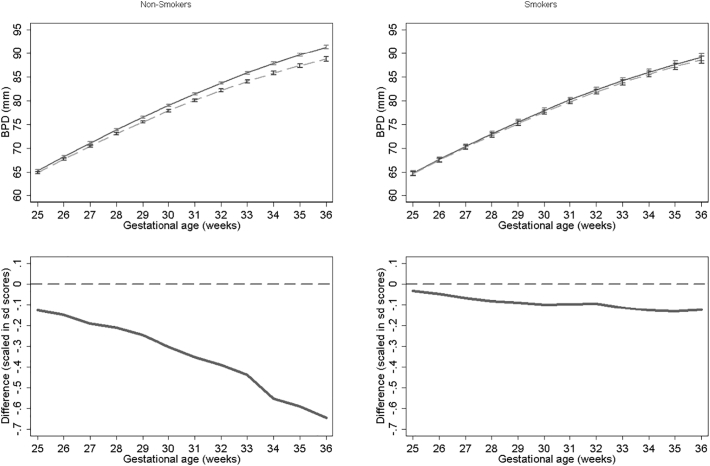
Estimated growth trajectories and relative differences for different levels of PM_10_ exposure for bi-parietal diameter stratified by maternal smoking status. Top panels show estimated growth trajectories for minimum exposure (solid lines) compared maximum exposure (dashed line). Bottom panels show the difference in these trajectories expressed in units of SD scores. The models are adjusted for parity, age, sex, smoking, social class, maternal height and weight. To aid interpretation, x axis is truncated below 25 weeks.

Results for BPD models stratified by smoking status for NO_2_, PM_2.5_, PM_10_ are presented in Fig. 6, Fig. 7, Fig. 8 respectively. None of the models for smokers show significant differences in growth across pregnancy. In contrast NO_2_, PM_2.5_ and PM_10_ models for non-smokers show significant differences from late second trimester onwards. At 36 weeks we estimate differences in BPD's of 1.79 mm, 2.38 mm and 2.59 mm's for NO_2_, PM_2.5_ and PM_10_ respectively for non-smokers. PM_10_ exposure among smokers shows the largest relative difference. In the full interaction model these differences and interaction terms were statistically significant (Supplementary Tables S1 and S2). Results were consistent in sensitivity analysis when stratifying by term births and fetal sex.

## 4 Discussion

This large population study examines the association between maternal exposure to ambient air pollutants PM_2.5_, PM_10_, and NO_2_ during pregnancy and fetal size and growth throughout the second half of pregnancy and at birth. We find that particulate matter (PM) (both PM_10_ and PM_2.5_) and NO_2_ are consistently related to head growth restriction from the 25th week of gestation onwards and occipotofrontal circumference at birth but that importantly these associations appear to be absent in smokers. We also observe small but insignificant differences in utero femur length and significant difference in crown heel length at birth. Though we are reporting annual rather than nine month averages, the ambient exposures in our region are notably lower than those in previous studies (Aguilera et al., 2010, Iñiguez et al., 2012, Ritz et al., 2014, van den Hooven et al., 2012).^1^ This suggests that the “safe” ambient air exposure for pregnant mothers may be lower than current levels laid out in Scottish National air quality objectives and the European Directive limit and target values for the protection of human health. These guidelines currently state that annual averages for PM_10_, PM_2.5_ and NO_2_ should not exceed 18, 10 and 40 μg/m^3^ respectively (DEFRA, 2015).

Though the study is an observational design, there are several reasons to support the assertion that the associations identified may be causal. Firstly, there are plausible pathways linking air pollution exposure to fetal growth restriction since PM (particularly PM_2.5_) and NO_2_ can cross from the inhaled air into the blood stream (Geiser et al., 2005) and can also cross the placenta and enter fetal circulation (Wick et al., 2010). Secondly, our results are internally consistent in that we observe reductions in head size and smaller reductions in femur length in late second trimester which are observed as significant differences in birth outcomes (crown heel length and occipito frontal circumference) but no associations with abdominal circumference. Furthermore, we show graded dose-response relationships across our exposure categories and demonstrate that the effects differ between smoking and non-smoking mothers.

Six previous studies have examined the effect of air pollution for in utero fetal growth using ultrasound image data and of these only three have examined PM_10_ and none have explored the effect of PM_2.5_which in our study showed the strongest and most consistent association with growth restriction. Generally, across all of these previous studies, there remains a lack of agreement. For example, in a cross-sectional analysis Hansen et al. (2008) report associations between increased PM_10_ exposure and reductions in abdominal circumference (AC), head circumference (HC) and femur length (FL) but only between pregnancy days 91–120. In contrast, a study of 500 pregnancies by Ritz et al. (2014) observed a reduction in BPD in association with increasing nitrogen oxide exposures but only in the 37th gestational week; there were no associations between PM_10_ exposure and BPD at 19, 29 or 37 weeks gestation. A third study of 7772 pregnancies found that increased exposure to NO_2_ and PM_10_ was associated with reduced HC, FL and EFW during the third trimester (van den Hooven et al., 2012). Interestingly, despite finding associations with in utero growth, Van den Hooven et al. (2012) reported no association between pollution exposure and outcomes measured at birth implying a process of catch-up growth later in pregnancy. In contrast, we observed growth restriction associated with pollutant exposure both in utero from approximately 25 weeks onwards and at birth for birthweight, crown heel length and head circumference.

One explanation for the difference between our study and that of Van den Hooven et al. (2012) could be that the latter use cubic splines to model changes in standard deviation scores across pregnancy which are often prone to erratic behaviour at each end of the available data whereas our study uses fractional polynomial functions which are less prone to artefactual curve shapes (Tilling et al., 2014). Additionally, different average ambient exposures as well as differences in the composition of different pollutants between studies may partly explain the apparently inconsistent findings and differences in sample size may mean that some studies were underpowered.

The use of fetal ultrasound measurements has given insight into associations between maternal environmental exposure during pregnancy and reduced growth (Aguilera et al., 2010, Carvalho et al., 2016, Hansen et al., 2008, Iñiguez et al., 2012, Malmqvist et al., 2017, Ritz et al., 2014, Slama et al., 2009, van den Hooven et al., 2012). Two studies from the INMA cohort (Aguilera et al., 2010, Iñiguez et al., 2012) have showed associations between NO_2_ and aromatic hydrocarbon exposure and a variety of fetal measurements indicating that exposure mainly effects growth later in pregnancy, especially for head size. There are other exposures beyond air pollution that are also linked with restricted fetal growth including maternal alcohol consumption which is associated with restricted third trimester BPD (Kfir et al., 2009) and prenatal exposure to bisphenol A which is associated with progressively marked reductions in HC as pregnancy develops (Snijder et al., 2013).

Our observation that the effects of ambient air exposure was subsumed by maternal smoking is consistent with the notion that cigarette smoke acts as a higher dose equivalent of ambient outdoor particulate exposure (Pope et al., 2009). Particle sizes in cigarette smoke are typically smaller than 1 μm and therefore penetrate into the body to a similar degree as PM_2.5_ ambient pollution. This includes crossing into maternal blood in the lungs, crossing the placenta and causing blood vessel inflammation. Despite this, few previous in utero ultrasound studies have examined whether the relationship between ambient air pollution exposure and fetal growth is modified by smoking status. Our observation that, in relative terms, the effect of ambient particulate exposure appears to be significantly reduced among smoking mothers is therefore noteworthy.

One explanation may be that that the dose response function for pollutant exposure and fetal growth is non-linear (Winckelmans et al., 2015). Given that the fetal environment of a smoking mother is known to be hazardous for the developing fetus, it may be the case that additional exposure to air pollution may not result in additive growth restriction in an already compromised fetal environment. Evidence from other health outcomes supports this interpretation. For cardiovascular mortality, it has been observed that effect estimates calculated at lower pollutant exposures result in implausibly large mortality risks when linearly extrapolated to estimate risks associated with higher pollutant exposures (Ostro, 2004). More recently, for cardiovascular mortality, Pope et al. (2009) demonstrated the effects of different dose levels of PM_2.5_ including those typical of ambient outdoor environments, environmental and second hand tobacco smoke and active smoking. The results suggested sharp increases in mortality at the lower doses which flattened off at concentration doses typical of active smoking. Similar effects were observed for asthma exacerbation (Rabinovitch et al., 2011) but not lung cancer (Pope et al., 2011, Turner et al., 2014). In terms of the shape of the exposure response curve our study therefore appears to suggest that the effect of PM_2.5,_ for in utero fetal head growth, may be of a similar shape (i.e. greater increase in effects associated with pollutant increase at lower exposures) to that of CVD mortality which might be expected given the importance of the cardiovascular system for ensuring a protective in utero environment and healthy placenta. Alternatively, it has been shown that tobacco use during pregnancy is associated with reductions in the risk of hypertension and preeclampsia which are themselves associated with growth restriction (Wikström et al., 2010) and so one possibility is that this effect among smokers subsumes the growth restriction effect of air pollution. Because smoking during pregnancy is associated with other factors such as alcohol consumption and poor diet, it is also possible that the effects we observe may be a result of not just smoking but these other factors as well.

From a public health policy perspective, the smoking air pollution interaction effect poses an interesting question. Population attributable risks associated with air pollution exposure are far higher given that potentially the entire population is exposed (Smith and Peel, 2010). Furthermore, although reducing exposure on an individual basis is difficult given the ubiquity of high pollution concentrations in particularly urban environments, our findings appear to support the argument that even small reductions at the lower end of the exposure range may yield significant gains (Smith and Peel, 2010). Moreover, if smoking subsumes the effect of pollutant exposure, omitting a smoking interaction term will mask some of the pollution effect in the non-smoking population raising the possibility that previous studies may have underestimated pollution effect sizes in non-smoking pregnant women. Furthermore, if we consider the effects of second-hand smoke, the differences we have observed may be larger than our analysis suggests. Because we were unable to measure exposure to second-hand smoke, our group of non-smoking mothers is likely to include those exposed to tobacco smoke from other smokers in the household. If these mothers were included in the group of smoking mothers the resulting differences in the effect of pollution exposure are likely to be greater. Finally, our findings highlight the possibility that current guideline concentration values may be too high. In 2005, the WHO recommended as safe annual mean concentration values below 10 μg/m^3^, 20 μg/m^3^ and 40 μg/m^3^ for PM_2.5_, PM_10_ and NO_2_ respectively. Our study demonstrates fetal growth restriction effects that are not markedly different to the effects of smoking at ambient concentration levels that are less than these ‘safe’ threshold values.

The study has a number of strengths. Perhaps most importantly, it is the first study to relate PM_2.5_ exposure to a fetal ultrasound cohort. Secondly, the study draws on routinely collected maternity birth and ultrasound data which provided us with full population coverage and allowed us to estimate growth curves from data points from all points of gestation age. It is also the largest study to examine the effect of air pollution for in utero growth and allowed us examine models stratified by smoking status. Finally, the study was conducted in an environment with relatively low level of air pollution; the highest values for particulates were only twice the mean values indicating that expoures were persistently “low”. Our findings are therefore important because, despite these persistently low exposures, we still observe significant fetal growth restriction which, from a public health and population level, should be of concern given the ubiquity of air pollution and the challenges that are associated with reducing exposure, particularly in urban areas. Methodologically the study has a number of strengths including the use of fractional polynomial models which allow for more flexible modelling of growth in conjunction with ultrasound scan information covering the entire gestational period. We also used spatially detailed pollution information, which we and others (Butland et al., 2013, Dibben and Clemens, 2015) have noted elsewhere is preferable to using the nearest static monitor to assign exposure which is likely to introduce a bias towards the null hypothesis.

The study has some limitations. Firstly, we conduct multiple statistical tests which increases the possibility of observing findings on the basis of chance. However, in comparison to previous studies we find a high degree of consistency in our results. Secondly, we relied on annual average pollution information which did not vary across pregnancy and thus were not able to examine the effects of higher or lower exposure at different points during gestation. Thirdly, third trimester measurements are not routinely collected and there is a chance that scans at later gestational ages may not be a random sample with respect to growth restriction. They may contain proportionally higher numbers of foetuses that are smaller on average resulting in overestimation of the population prevalence of growth restriction later in pregnancy. We mitigated this in the models by examing relative growth and in general we argue that the selective provision of third trimester scans is unlikely to alter the conclusions significantly. The most likely effect will be to produce a bias towards the null hypothesis because the contrast between exposed and unexposed foetuses will likely contain a smaller range of fetal sizes as it is restricted to those missing information for healthier pregnancies that did not receive a third trimester scan. Furthemore, air pollution exposure itself is unlikely to increase the probability of receiving a third trimester scan independently of the hypothesised growth restricting effect of interest. Thus, pollutant exposure is random though its effects may increase the probability of receiving a third trimester scan. This conclusion is plausible when considering that the effects for birth outcomes (which are estimated from information on all pregnancies and not just those with a third trimester scan) remain consistent with the effects observed in utero. Fourthly, we were unable to consider other potential confounders including maternal exposure to road traffic noise, temperature, diet, occupational exposure, pesticides and paint during pregnancy which may be associated jointly with air pollution and the fetal growth. We were also restricted to considering only PM_2.5_, PM_10_ and NO_2_ and more studies are needed to consider more source specific exposures. We were also unable to consider exposure to second-hand smoke. A final limitation is that we were unable to determine more detailed maternal activity patterns beyond simple residential location and thus exposure estimates will suffer some misclassification. However, previous studies incorporating work place location have showed little difference in final model estimates (Dibben and Clemens, 2015) and much of the mismeasurement is likely to be random in terms of pollution exposure.

## 5 Conclusions

The results from this large population cohort of ultrasound scans presents perhaps the strongest evidence to date of a growth restricting effect of air pollution for fetal BPD growth during the period towards the end of second trimester onwards. Furthermore, our observation that these effects are restricted to non-smoking mothers supports the hypothesis that pollutant exposure response curves may be non-linear with the greatest harms accrued at lower exposures. In terms of policy implications, the findings suggest the need to focus efforts on reducing exposures at the lower end of the pollutant scale. Furthermore, given the relatively low exposure levels in our study area, existing guidelines on safe concentration thresholds may currently be too high with respect to the potential effects for pregnancy. Finally, future research should focus on the post-natal and beyond consequences of air pollution related head growth restriction, for example how this impacts educational attaintment and employment status of offspring.

## Competing financial interests

The authors declare they have no actual or potential competing financial interests.

## Appendix A Supplementary data

Supplementary material Image 1

## Footnotes

1 For PM_10_ we report a mean of 13 μg/m^3^ (sd 2.29) compared to between 39 and 41 μg/m^3^ (sd 6–9) and 30 μg/m^3^ and for NO_2_ we report a mean of 14 (sd 8.24) compared to 32 μg/m^3^ (sd: 11), 37–39 μg/m^3^ (sd: 8–9) and 40 μg/m^3^ in previous studies.
