# Supplementary material for: Maternal exposure to ambient air pollution and fetal growth in North-East Scotland: A population-based study using routine ultrasound scans
Source: Environ Int. 2017 Oct;107:216–26. doi: 10.1016/j.envint.2017.07.018 (PMC5571229; doi:10.1016/j.envint.2017.07.018)
Supplement: Supplementary file 1 — Supplementary material [file mmc1.docx]

**Supplementary Tables**

| **Table S1: Mixed effects model showing BPD growth trajectories associated with covariates and with a three way interaction between smoking, gestational age terms and particulates less than 2.5 microns. Pollution coefficients are scaled in units of 5 µg/m^3^ and gestational age in weeks.** | | | |
| --- | --- | --- | --- |
| **Variable** | **Coefficient** | **95% CI** | |
| **Smoker*PM_25_*Gestational age 1** | .0135548*** | .0034844 | .0236252 |
| **Smoker*PM_25_*Gestational age 2** | -.0040257*** | -.0067244 | -.001327 |
| **Non-smoker*PM_25_*Gestational age 1** | .0092019* | -0.0007544 | .0191593 |
| **Non-smoker*PM_25_*Gestational age 2** | -.0026966** | -.0053637 | -.0000294 |
| **Fetal sex*Gestational age 1** | .024775*** | .0181575 | .0313924 |
| **Fetal sex*Gestational age 2** | -.0064631*** | -.0082363 | -.0046899 |
| **Maternal weight*Gestational age 1** | -.0003028*** | -.0005208 | -.0000848 |
| **Maternal weight*Gestational age 2** | .0000952*** | .0000369 | .0001535 |
| **Gestational age 1** | .4468244*** | .4247647 | .468884 |
| **Gestational age 2** | -.1050098*** | -.1109111 | -.0991084 |
| **PM_25_** | -.6461571 | -1.429524 | .1372098 |
| **Parity**  **(ref: One previous pregnancy)** |  |  |  |
| **Nulliparous** | .2603916*** | .1513 | .3694832 |
| **Two previous pregnancies** | -.0401439 | -.2059178 | .1256301 |
| **Three or more pregnancies** | -.2504849** | -.4827086 | -.0182612 |
| **Maternal age**  **(ref: 20-40)** |  |  |  |
| **19 and under** | -.2040478** | -.4328632 | .0247675 |
| **Over 40** | .508607*** | .1455643 | .8716498 |
| **Fetal sex**  **(ref: female)** | -1.058683*** | -1.563169 | -.5541972 |
| **Maternal Height** | .0303177*** | .0222545 | .0383809 |
| **Maternal Weight** | .0038257 | -.0131913 | .0208426 |
| **Maternal smoking**  **(Ref: Non-smoker)** | -.2353583 | -.6584394 | .1877228 |
| **Maternal Social class**  **(Ref: Professional occupations)** |  |  |  |
| **Managerial and technical occupations** | -.0271877 | -.1861228 | .1317473 |
| **Skilled non-manual occupations** | .0781185 | -.1058841 | .262121 |
| **Skilled manual occupations** | .0185043 | -.1856676 | .2226763 |
| **Partly-skilled occupations** | -.0696628 | -.2761922 | .1368665 |
| **Unskilled occupations** | .1098355 | -.2367516 | .4564226 |
| **Year of scan** |  |  |  |
| **2003** | .1853345** | .0319889 | .33868 |
| **2004** | .1574403** | .0080767 | .3068039 |
| **2005** | -.1988887** | -.3846008 | -.0131765 |
| **2006** | .0089359 | -.1856998 | .2035717 |
| **2007** | .5315663 | -.2658518 | 1.328984 |
| **2008** | 1.530907** | .2324767 | 2.829337 |
| **2009** | -.2487157 | -1.492546 | .9951141 |
| **2010** | -16.88186*** | -20.98686 | -12.77686 |
| **2011** | -8.661979*** | -12.76654 | -4.557416 |

| **Table S2: Mixed effects model showing BPD growth trajectories associated with covariates and with a three way interaction between smoking, gestational age terms and particulates less than 10 microns. Pollution coefficients are scaled in units of 10 µg/m^3^ and gestational age in weeks.** | | | |
| --- | --- | --- | --- |
| **Variable smoking interaction** | **Coefficient** | **95% CI** |  |
| **Smoker*PM_10_*Gestational age 1** | .0155679** | .0004322 | .0307036 |
| **Smoker*PM_10_*Gestational age 2** | -.0047763** | -.0088316 | -.000721 |
| **Non-smoker*PM_10_*Gestational age 1** | .0109109 | -.004167 | .0259888 |
| **Non-smoker*PM_10_*Gestational age 2** | -.0033218 | -.0073606 | .0007171 |
| **Fetal sex*Gestational age 1** | .0248714*** | .0182391 | .0315037 |
| **Fetal sex*Gestational age 2** | -.0064904*** | -.0082677 | -.0047131 |
| **Maternal weight*Gestational age 1** | -.000315*** | -.000534 | -.0000961 |
| **Maternal weight*Gestational age 2** | .0000983*** | .0000398 | .0001569 |
| **Gestational age 1** | .4590643*** | .4420077 | .4761208 |
| **Gestational age 2** | -.1085257*** | -.1130891 | -.1039624 |
| **PM_10_** | -.4986663 | -1.60034 | .6627018 |
| **Parity**  **(ref: One previous pregnancy)** |  |  |  |
| **Nulliparous** | .2585827*** | .1492443 | .3679211 |
| **Two previous pregnancies** | -.0426739 | -.2089569 | .1236091 |
| **Three or more pregnancies** | -.2346933** | -.4675393 | -.0018473 |
| **Maternal age**  **(ref: 20-40)** |  |  |  |
| **19 and under** | -.2152695** | -.4449782 | .0144393 |
| **Over 40** | .5152437*** | .152391 | .8780963 |
| **Fetal sex**  **(ref: female)** | -1.059219*** | -1.564621 | -.553817 |
| **Maternal Height** | .0307377*** | .0226611 | .0388143 |
| **Maternal Weight** | .0048485 | -.0122275 | .0219245 |
| **Maternal smoking**  **(Ref: Non-smoker)** | -.3777519 | -.6310782 | -.1244256 |
| **Maternal Social class**  **(Ref: Professional occupations)** |  |  |  |
| **Managerial and technical occupations** | -.0260286 | -.1850814 | .1330243 |
| **Skilled non-manual occupations** | .0722029 | -.1121047 | .2565105 |
| **Skilled manual occupations** | .0250541 | -.1792348 | .2293431 |
| **Partly-skilled occupations** | -.0779948 | -.2851869 | .1291974 |
| **Unskilled occupations** | .0788307 | -.2694366 | .427098 |
| **Year of scan** |  |  |  |
| **2003** | .112198* | -.0305231 | .2549192 |
| **2004** | .0882381** | -.058518 | .2349941 |
| **2005** | -.0453503 | -.1896293 | .0989286 |
| **2006** | .068339 | -.1213373 | .2580153 |
| **2007** | .6737502 | -.1100988 | 1.457599 |
| **2008** | 1.639255** | .3446734 | 2.933837 |
| **2009** | -.2277269 | -1.471313 | 1.015859 |
| **2010** | -16.93571*** | -21.04016 | -12.83126 |
| **2011** | -8.71659*** | -12.82137 | -4.611806 |
